# Effects of imperatorin on apoptosis and synaptic plasticity in vascular dementia rats

**DOI:** 10.1038/s41598-021-88206-7

**Published:** 2021-04-21

**Authors:** Ying Huang, Xiangping Liao, Huaiwei Wang, Jianghong Luo, Shanquan Zhong, Ziliang Zhang, Fang zhang, Jianping Chen, Fuhua Xie

**Affiliations:** 1 Key Laboratory of Prevention and Treatment of Cardiovascular and Cerebrovascular Diseases, Ministry of Education, Gannan Medical University, Ganzhou, 341000 Jiangxi China; 2 Department of Neurology, the First Affiliated Hospital of Gannan Medical University, Ganzhou, 341000 Jiangxi China; 3 Gannan Branch Center of National Geriatric Disease Clinical Medical Research Center, Gannan Medical University, Ganzhou, 341000 Jiangxi China; 4 Department of Psychology, The Third People’s Hospital of Ganzhou, Ganzhou, 341000 Jiangxi China; 5 Department of Neurology, The Second People’s Hospital of Mengcheng, Bozhou, 233500 Anhui China; 6 Department of Preventive Medicine, Basic Medicine School, Gannan Medical University, Ganzhou, 341000 Jiangxi China; 7 Gannan Medical University, Ganzhou, 341000 Jiangxi China; 8 Department of General Practice, Ganzhou People’s Hospital, Ganzhou, 341000 Jiangxi China; 9 School of Basic Medicine, Gannan Medical University, Ganzhou, 341000 Jiangxi China

**Keywords:** Drug discovery, Neuroscience, Neurology

## Abstract

In view of the complicated pathophysiological process of vascular dementia (VD), drugs for the clinical treatment of VD mainly target related risk factors, while drugs with excellent efficacy in cognitive function are still relatively lacking. Imperatorin (IMP), an active constituent extracted from angelica dahuricae and notopterygium Notopterygii, which has anti-inflammatory, vasodilator, anticoagulant, block calcium channel, anticonvulsant, and anti oxygen free radical injury properties. Therefore,the present study examined its effects on VD rats and the underlying molecular mechanisms, in order to provide promising therapeutic methods. VD was established by modified ligation of perpetual two-vessel occlusion (2VO). After 2VO surgery, IMP (2.5, 5, and 10 mg/kg) was administered by intraperitoneal injection for 12 consecutive weeks to evaluate therapeutic effects. Cognitive function was verified by the Morris water maze. The neuronal morphological changes were examined via Hematoxylin–Eosin staining. Real-Time PCR and Western blot were used for detecting pro- and antiapoptotic biomarkers, and the hippocampus synaptic damage was examined by Transmission electron microscope. We revealed that 2VO-induced cognitive impairment, hippocampus CA1 neuron damage, apoptosis and synaptic damage. IMP-treatment significantly improved 2VO-induced cognitive deficits and hippocampus neuron damage. Molecular analysis revealed that IMP inhibited apoptosis through the down regulation of Bax, Caspase-3 and upregulation of Bcl-2. Meanwhile, IMP-treatment markedly improved synaptic ultrastructure morphology, increased the SAZ length, PSD thickness and up-regulated PSD-95 expression. Collectively, our findings demonstrated that IMP was effective in the treatment of 2VO-induced VD via inhibiting apoptosis of hippocampus neurons and reducing the synaptic plasticity destroy.

## Introduction

Imperatorin (IMP) [9-[(3-methylbut-2-en-1-yl)oxy]-7H-furo[3,2-g] chromen-7-one] is a kind of Furanocoumarinone, which is one of the effective components of Fructus Cnidii, angelica dahuricae and notopterygium Notopterygii^1^. Lots of experimental researches have confirmed that natural coumarins and their derivatives are a new type of neuroprotective agents, which have antioxidant activity, inhibit and scavenge ROS production, and reduce the damage caused by oxygen free radicals^2^. As well as, imperatorin possess anti-inflammatory, vasodilator, anticoagulant, platelet aggregation inhibition, antithrombotic, block calcium channel, anticonvulsant, and anti oxygen free radical injury properties^3–6^.We also found imperatorin can improve the learning and memory function in vascular dementia model mice. As we all know, chronic cerebral hypoperfusion(CCH) caused by various reasons makes the central nervous system lacking of glucose and oxygen supply, leading to neuron damage, which is the pathophysiological process of vascular dementia(VD), involving oxidative stress injury, chronic inflammatory response, cholinergic nerve injury, cell apoptosis, autophagy, synaptic damage and other cellular and molecular biological reactions^7–11^. In view of their close relationship, more and more scholars pay heed to the potential mechanism of CCH induced VD in order to provide promising therapeutic methods.

CCH induced apoptosis plays a critical role in the development of VD. Apoptosis has three stages: signal transduction, gene regulation and apoptosis effect. Bcl-2 and Bax are essential regulatory factors in Bcl-2 family^12^. Caspase-3 is the most principal protease in apoptosis signal transduction pathway. When cerebral ischemia occurs, the proapoptosis protein Bax rapidly changes its conformation in response to the apoptosis signal, translocates from the cytoplasm to the outer membrane of mitochondria, forms protein dimer with anti apoptosis protein Bcl-2, regulates mitochondrial permeability, releases some small molecules such as cytochrome c, enters the cytoplasm, binds with apoptosis protease activating factor and activates in sequence Caspase-9 and Caspase-3 can induce apoptosis^13,14^.

In addition, damage of synaptic plasticity induced by CCH has also been confirmed to be one of the pathogenesis of VD, especially the morphological structure and functional state of synapses, which are closely related to the cognitive impairment of VD^15^. Synapses are the contact points of structures and functions between neurons, and the plasticity of synapses is the neurobiological basis of learning and memory^16^. Studies have shown that post synaptic density (PSD) plays a key role in the regulation of synaptic function and synaptic signal transduction mechanism. A layer of dense substance under the post synaptic membrane contains more than 30 protein components closely related to synaptic transmission, such as calmodulin and PSD-95. Among them, PSD-95 is an important structure to realize the transmission and integration of synaptic signals, which is linked with the progress of cerebral ischemia injury, dementia and other diseases^17^. On the one hand, PSD-95 can aggregate N-methyl-D-aspartate receptor (NMDAR), advance the formation of long-term potentiation (LTP), and mediate the process of learning and memory^18,19^. On the other hand, PSD-95 can also bind to nonreceptor tyrosine protein kinases and participate in the transmission of neurotoxic signals mediated by NMDAR over expression^20^. When cerebral ischemia and hypoxia, the homeostasis of the body's internal and external environment, such as energy metabolism disorder, the accumulation of reactive oxygen species, the pathological increase of glutamate, not only cause the changes of synaptic morphology and neurotransmitters, but also affect the function of PSD95 integrated synaptic signal, the point-to-point transmission between synaptic contacts and the formation of LTP, which eventually leads to the decline of learning and memory ability^21,22^.

Wang et al.^23^ found that IMP can reverse the expression of apoptosis genes Bcl-2 and Bax through the mitochondrial pathway, and reduce the apoptosis of oxygen glucose deprivation reperfusion cell model. In vivo, IMP can decrease the size of cerebral infarction and promote the recovery of neurological function in mice with cerebral ischemia–reperfusion injury. In addition, it was published by Chowdhury et al.^24^ that IMP can improve the learning and memory impairment induced by lipopolysaccharide by reducing pro-inflammatory cytokines and regulating brain-derived neurotrophic factors.

Object to investigate whether IMP has a neuroprotective effect on VD mice and its possible mechanisms. In this study, modified bilateral common carotid artery occlusion was performed, and morris water maze was used to assess whether IMP can improve cognitive function of VD mice. Meanwhile, effects of IMP on hippocampus cell apoptosis and synaptic plasticity of VD mice were detected to reveal the possible molecular mechanisms, which laid a theoretical foundation for the application of IMP in the treatment of VD and provided new ideas for drug therapies.

## Materials and methods

### Animals and treatment

This animal experiments were performed according to the ethical guidelines of Gannan Medical University Animal Ethics Committee and the National Institutes of Health. And the study was carried out in compliance with the ARRIVE guidelines.Healthy, 80 adult male Sprague–Dawley (SD) rats aged 8 weeks were housed with a 12 h light–dark cycle at room temperature (19 ± 2 °C) and free access to food and water.

**Figure 1:**
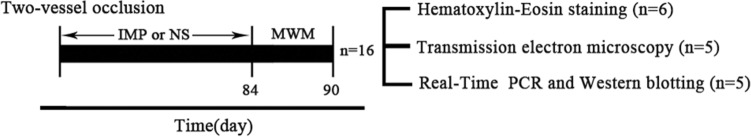
The schematic diagram of the experimental design. IMP = Imperatorin. NS = normal saline. MWM = Morris Water Maze.

After administration of 1% Pentobarbital sodium (40 mg/kg, i.p.) for anesthesia, the common carotid artery was separated from the adjacent vagus nerve and double-ligated with 6–0 silk sutures to perform the two-vessel occlusion (2VO). Sham-operated rats were subjected to the same procedure, except for the carotid ligation. The schematic diagram of the experimental design is depicted in Fig. 1. 80 animals were randomly divided into the following groups: group 1 (n = 16) (sham group with no occlusion), group 2 (n = 16) (control group with 2VO procedure), group 3 (n = 16) (2VO group with IMP-2.5 administration), group 4 (n = 16) (2VO group with IMP-5 administration), and group 5 (n = 16)(2VO group with IMP-10 administration)^23^. Rats received 2VO and were administered saline or IMP the first day after surgery. IMP (2.5, 5, 10 mg/kg) was administered intraperitoneally (i.p.) in treatment with a 12 weeks.IMP (powder, purity 98%) was purchased from Aifa (ChengDu, China). IMP was dissolved indimethylsulfoxide (DMSO) (12.5%), Tween 80(2%), normal saline (NS) and administered by intraperitoneal injection.

### Morris water maze

The Morris water maze (MWM) is one of the most definitive test methods for detecting rodent's cognitive function, especially for testing hippocampus-related brain space learning and memory. 24 h after the last treatment, all rats began the 5-day MWM test. The device is composed of a circular pool, which was 150 cm in diameter and filled with water and latex liquid at 25 ± 1 °C to a depth of 50 cm, divided into four quadrants, I, II, III and IV, and with the equal distance to the edges. During the acquisition training, the hidden black platform (10 cm in diameter) was located in the center of quadrant III and submerged 2 cm below the water surface. The rats were first subjected to the positioning and navigation test for 5 consecutive days, and four trials per day. In each trial, the rats were gently released into the water by forcing the tank wall at four different quadrants around the pool. The rats were paid a maximum of 120 s to find the hidden black platform. When reaching the platform, the rats were allowed to stay on it for 10 s. If the rat failed to find the platform within 120 s, the training was terminated and we recorded the escape latency of this rat was 120 s. Then the rat was guided to the covert black platform and allowed to stay on the platform for 10 s. The latency to escape onto the hidden platform was recorded as the ability of spatial learning. To evaluate spatial memory, the space exploration trial was performed 1 day after the last training trial. In this trial, we removed the platform from the tank and the rats were permitted to swim freely for 120 s in the pool. The pool was situated in the center of a room containing prominent spatial cues and had a recessed video camera mounted on the ceiling to monitor swim paths. The video signal was relayed to a computer running specialized data acquisition software (‘Watermaze’ Actimetrics, Wilmette, IL, USA) that records the path made by the animal^25^.

### Hematoxylin–Eosin(HE) staining

After the behavioral test, 6 rats in every group were anesthetized with 1% Pentobarbital sodium (40 mg/kg, i.p.) and then perfused transcardially with 0.01% heparin/saline followed by 4% paraformaldehyde/0.1 mol/L phosphate buffer saline (PFA/PBS, pH7.4). The brains were removed and post-fixed in PFA solution for 12 h and then dehydrated by ethanol with different concentrations (75%, 85%, 95%, 100%), then xylene I, II, III were respectively 20 min, followed by dipping wax I, II 60 min, Each brain block containing the CA1 or striatum was cut into 4-μm-thick coronal sections on a microtome. We dehydrated with xylene, ethanol solutions of different concentrations, and distilled water, then stained with hematoxylin for 5 min, differentiate with 1% hydrochloric acid and alcohol for 30 s, and return to blue with 0.6% ammonia; rinsed with tap water between each step.Then the slice was stained by eosin solution for 2 min, after dehydration, mounted it with neutral resin, and finally we observed the morphological changes of the neurons in the hippocampus CA1 area along the dorsal/ventral axis by using an 400 × light microscope (Olympus, Tokyo, Japan) and a relatively constant number of pyramidal neurons from that was photographed. The number of cells in the same area from each photo was quantified, and the mean value of 3 sections was regarded as the number of surviving pyramidal neurons from that sample.

### Real-time PCR (RT-PCR)

In order to evaluate the neuroprotective effect of IMP on VD, after the behavioral test, 5 rats in each group were decapitated, and the brains were quickly removed, the hippocampus tissue was isolated, and quickly frozen with liquid nitrogen, and stored at -80℃ under for later use. For each sample, cDNA (2µL) was quantified and duplicated using SYBR Select Master Mix for CFX on a Light Cycler 480 II as per manufacturer instructions. Cycling conditions: 2 min at 50 °C, 2 min at 95 °C, 40 cycles of 15 s at 95 °C, 1 min at 60 °C. Melt curve cycles were immediately performed and the cycling conditions were as follows: 30 s at 95 °C, 15 s at 60 °C. Melt curve analysis was performed to verify primer specificity. Data are displayed as a fold change above the proliferative condition mRNA levels using 2^−∆∆Ct^ values^26^. Primer design and synthesis all target genes Bax, Bcl-2, Caspase-3 and β-Actin sequences are derived from the database (https://pubmed.ncbi.nlm.nih.gov/), and primer design and synthesis are carried out by Shenggong(Shanghai, China).

Primer sequences:

- Bax
- Forwardprimer 5′-AAGAAGCTGAGCGAGTGTCTC-3′
- Reverseprimer 5′-ATGGTTCTGATCAGCTCGGG-3′
- Bcl-2
- Forwardprimer 5′-CAGCATGCGACCTCTGTTTG-3′
- Reverseprimer 5′-CTCACTTGTGGCCCAGGTAT-3′
- Caspase-3
- Forwardprimer 5′-GGAGCTTGGAACGCGAAGAA-3′
- Reverseprimer 5′-ACACAAGCCCATTTCAGGGT-3′
- β-actin
- Forwardprimer 5′-TGGAGCAAACATCCCCCAAA-3′
- Reverseprimer 5′-TGCCGTGGATACTTGGAGTG-3′

### Western blotting

The hippocampal tissues removed from the storage at  − 80 °C, which were dissolved with RIPA lysis buffer containing protease inhibitor cocktail and phosphatase inhibitor cocktail. The lysate was centrifuged at 12,000 g for 10 min, and the supernatant was used for analysis. Protein concentrations were determined with the BCA Protein Assay Kit (Thermo Fisher Scientific, United States). A total of 25 μg of protein was separated using 10% or 12% SDS polyacrylamide gels and transferred onto PVDF membranes. After being blocked with 5% skimmed milk for 1 h, the membranes were incubated with primary antibodies overnight on shaker. The primary antibodies including rabbit-anti-Bax (diluted at 1:1000), rabbit-anti-Bcl-2 (diluted at 1:2000), and rabbit-anti-PSD-95 (diluted at 1:2000) were purchased from Abcam (Cambridge, UK), rabbit-anti-Caspase-3 was obtained from Cell Signaling Technology(diluted at 1:1000, Beverly, MA, USA), and rabbit-anti-β-actin(diluted at 1:1500, Solarbio, Beijing, China) was used as a loading control. After being washed in Tris-bufered saline, the membranes were incubated with horseradish peroxidase-conjugated anti-rabbit secondary antibody (diluted at 1:1500, Abcam, Cambridge, UK). Cross reactivity was visualized using ECL (Amersham Pharmacia Biotech, Piscataway, NJ, USA) western blotting detection reagents and then analyzed through scanning densitometry by a Lab image system^25^.

### Transmission electron microscopy (TEM)

After the behavioral test, 5 rats in every group, About 1 mm × 1 mm × 1 mm pieces were rapidly cut from hippocampus in brain, fixed in 2.5% glutaraldehyde in 0.1 M phosphate buffer (PB) (pH 7.4) for 2 h at room temperature and then post-fixed in 1% osmium tetroxide in 0.1 MPB. Then, dehydrated in graded ethanol and embedded in beam capsules. Sections, 50–70 nm-thick, were cut from the embedded tissue and collected onto grids to air dry overnight. Stained grids with 2% uranyl acetate for 30 min and lead citrate for 15 min, and then observed under Transmission Electron Microscope (JEOL, JEM 1400 PLUS)^27^.

### Statistical analysis

Statistical analysis was performed using SPSS version 18.0 software for Windows (SPSS Inc., Chicago, IL, USA). The results are presented as the mean ± standard deviation(SD). One-way analysis of variance (ANOVA) is followed by the Student–Newman–Keuls test was used to compare differences between mean values for data involving more than two groups. For behavioral experiments, the results were analysed by two-way repeated-measures analysis of variance (ANOVA). Signifcance was set at *P* < 0.05.

### Ethical approval

All animal procedures were subject to approval by the Ethics Committee of Gannan Medical University and were performed in accordance with the rules of the Care and Use of Laboratory Animals. This article does not include any studies with human participants performed by any of the authors.

## Results

### IMP attenuates learning and memory impairments in the 2VO rats

**Figure 2:**
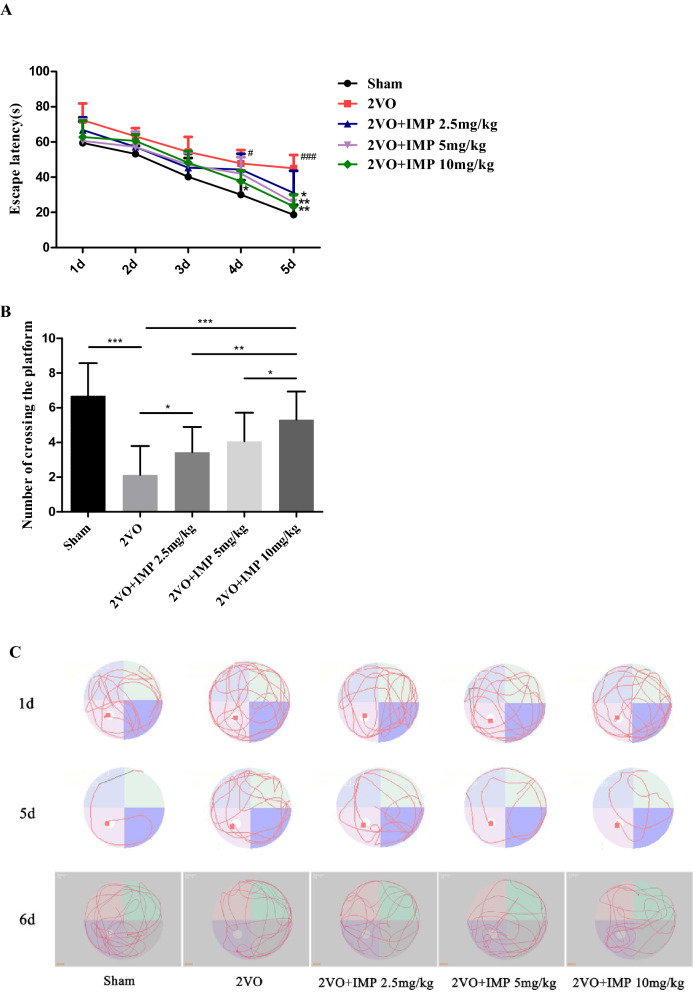
IMP attenuates learning and memory impairments in the 2VO rats. (**A**) Escape latency in each groups, #*p* < 0.05, ###*p* < 0.001 Sham group versus 2VO group and *p < 0.05, **p < 0.01, ***p < 0.001 2VO + IMP 2.5, 5, 10 mg/kg group versus 2VO group. repeated measured two-way ANOVA followed by Dunnett’s multiple comparisons test. (**B**) Number of crossing the platform in each groups, *p < 0.05, **p < 0.01, ***p < 0.001. (**C**) Trajectories of the rats in each group looking for the platform. n = 16 rats in each group.

We used the MWM test to measure the cognitive function of IMP treatment. The escape latency time was recorded during the 5 days for training trial. As shown in Fig. 2A, the escape latency time of animals in 2VO group was significantly more than that of sham group (*P* < 0.01), demonstrating the impairment of the cognitive function representing in VD models. However, treatment with IMP significantly ameliorated the poor behavioral ability in the MWM test (*P* < 0.05). Subsequently, a probe trial was performed on the sixed days to measure the spatial memory ability among different groups. Results indicated that rats in 2VO group presented worse memory ability with the number of crossing platforms than that of Sham group (*P* < 0.05). However, IMP (2.5, 5, and 10 mg/kg, respectively) treatments significantly improved the cognitive function impairment (Fig. 2B, *P* < 0.05). Furthermore, in our research, at the beginning of the positioning navigation experiment, the trajectories of the rats in each group looking for the platform were random and marginal. With the extension of the training time, the trajectories of the Sham and IMP treatment groups looking for the platform are purposeful and trendy, while the movement trajectories of the VD group until the end of the training are still random and marginal (Fig. 2C).

### IMP attenuates neuronal damage in the hippocampus induced by 2VO

**Figure 3:**
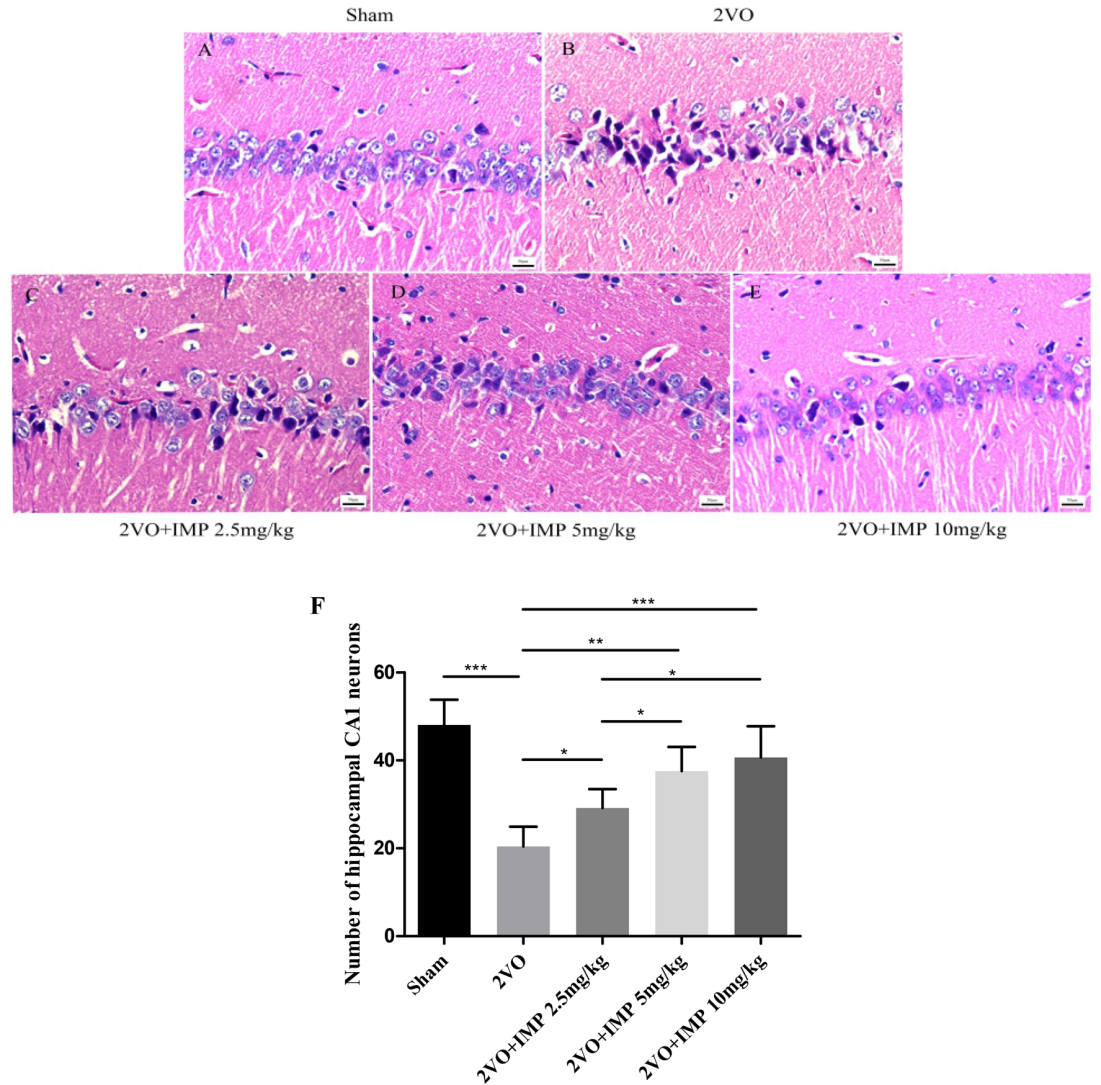
IMP attenuates neuronal damage in the hippocampus induced by 2VO. (**A**–**E**) Hematoxylin–Eosin (HE) stained hippocampus CA1 region from rats in each group. Scale bar = 50 μm. (**A**) Sham group, (**B**) 2VO group, (**C**) IMP 2.5 mg/kg group, (**D**) IMP 5 mg/kg group, (**E**) IMP 10 mg/kg group, (**F**) Number of hippocampal CA1 neurons of rats in each group (cells/ × 400 visual field). n = 4 rats per group. *p < 0.05, **p < 0.01, ***p < 0.001.

The hippocampal CAl area is highly sensitive to factors such as cerebral ischemia and hypoxia, which can easily induce delayed-type cell apoptosis, cause neurons in this area to be lost, and subsequently lead to a decline in learning and memory. Therefore, we chose the hippocampal CAl area to observe the damage of neurons in the hippocampus by ischemia and hypoxia. In this study, hippocampal CA1 region of different groups of rats was observed by HE staining (Fig. 3). The results showed that most neurons were lost, shrinkage, the arrangement was loose, the normal cell structure disappeared, the inner core area was deeply stained in the CA1 areas of the hippocampus in 2VO group compared with Sham group. However, IMP-treatment groups attenuated 2VO-induced.

neuronal damage, especially in the IMP (10 mg/kg) group. The above pathological changes were significantly improved, that is, the hippocampal cell morphological and structural damage was reduced, and its effect was dose-dependent. These changes were related to the improvement of the cognitive ability of the VD rat model.

### Effects of imperatorin on Bcl-2, Bax and Caspase-3 mRNA and protein expression of the 2VO rats

**Figure 4:**
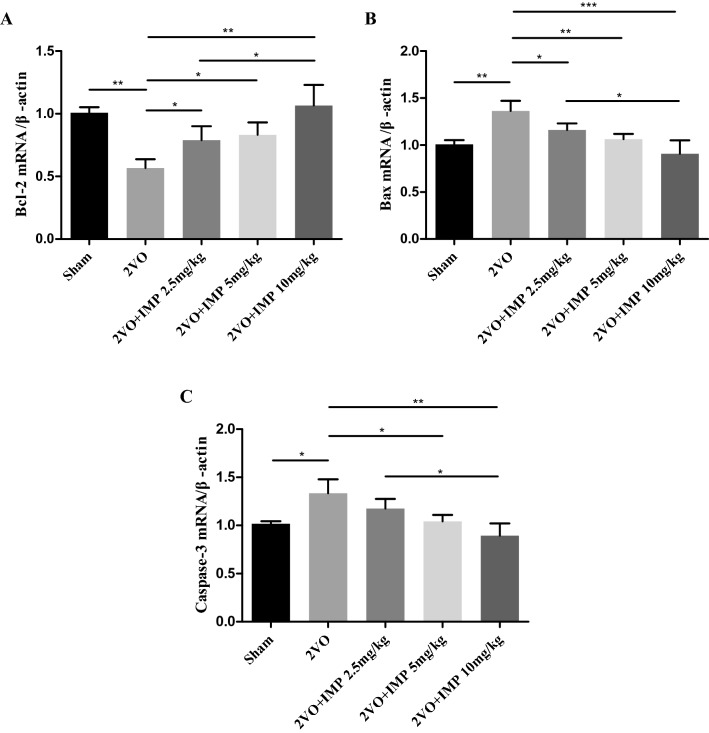
Effect of imperatorin on apoptosis-related genes expression of the 2VO rats. (**A**–**C)** Histograms showing mRNA relative expressions of Bcl-2 (**A**), Bax (**B**), Caspase-3 (**C**). n = 3 rats per group. *p < 0.05, **p < 0.01, ***p < 0.001 2VO + IMP 2.5, 5, 10 mg/kg group versus 2VO group.

**Figure 5:**
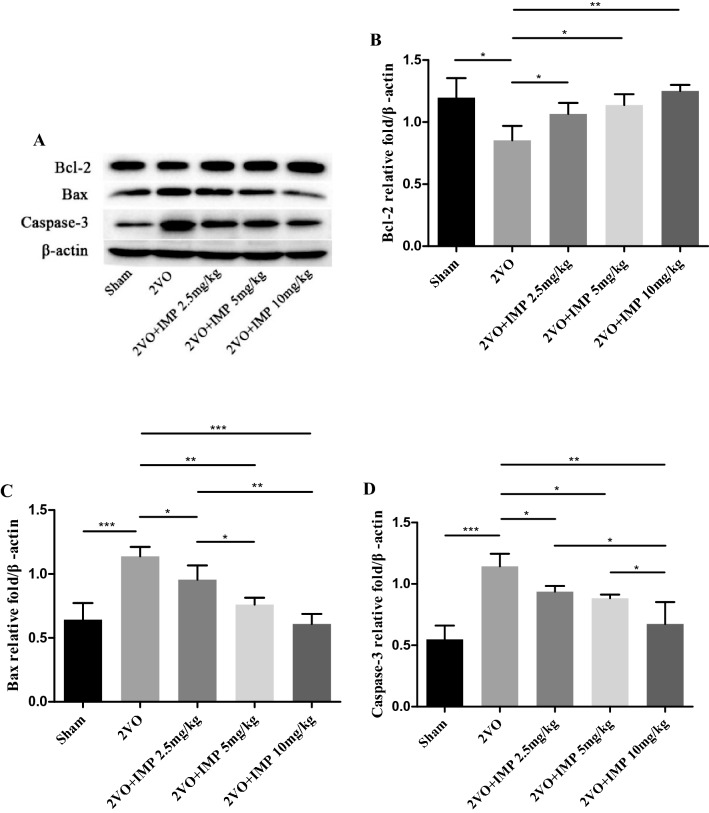
Effect of imperatorin on apoptosis-related proteins expression of the 2VO rats. (**A**) Western blot images representing the expression of Bcl-2, Bax, Caspase-3 and β-actin. (**B**–**D**) Histograms showing relative expressions of Bcl-2 (**B**), Bax (**C**), Caspase-3 (**D**). White space was used to make explicit for the grouping of blots cropped from different parts of the same gel or from different gels. Experiments were repeated three times and data are shown as mean ± s.d, n = 3 rats per group. *p < 0.05, **p < 0.01, ***p < 0.001.

As shown in Figs. 4 and 5, RT-PCR results and western blot analysis demonstrated that imperatorin treatment downregulated the mRNA and protein levels of the proapoptosis factor Bax (Figs. 4B and 5C) and its downstream factor Caspase-3 (Figs. 4C and 5D), upregulated the anti-apoptosis factor Bcl-2 (Figs. 4A and 5B) dose-dependently. These changes further supported our findings that imperatorin inhibited apoptosis caused by chronic ischemia and hypoxia and provided advantages to treatment of vascular dementia. IMP was able to inhibit the 2VO-induced apoptosis in a dose-dependent manner (*P* < 0.01).

### Effect of imperatorin on hippocampal synaptic ultrastructure and PSD-95 protein expression

**Figure 6:**
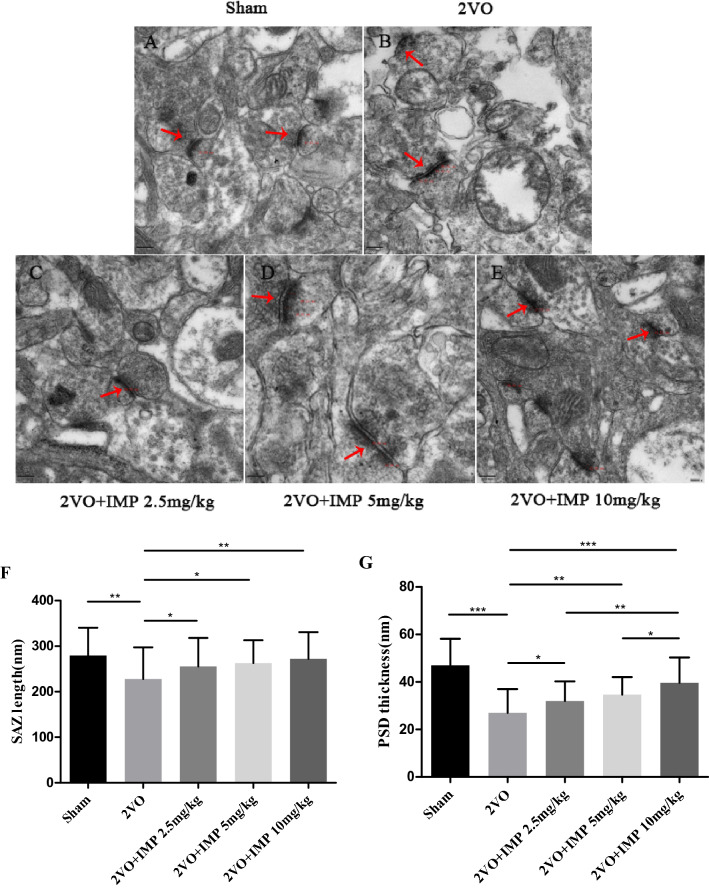
Effect of IMP on hippocampus synaptic ultrastructure. (**A**–**E**) Hippocampus CA1 region from rats in each group observed by Transmiss ion electron microscope (TEM) (× 50,000). Scale bar = 300 nm. (**A**) Sham group, (**B**) 2VO group, (**C**) IMP 2.5 mg/kg group, (**D**) IMP 5 mg/kg group, (**E**) IMP 10 mg/kg group, (**F**) SAZ length of hippocampus synaptic in each group, (**G**) PSD thickness of hippocampus synaptic in each group. n = 40 synapses. *p < 0.05, **p < 0.01, ***p < 0.001.

**Figure 7:**
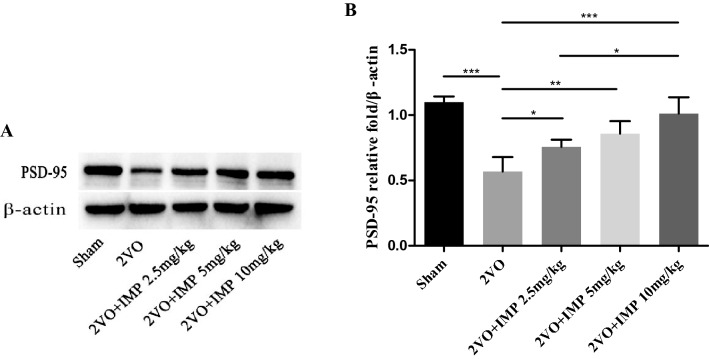
Effect of IMP on PSD-95 protein expression of the 2VO rats. (**A**) Western blot images representing the expression of PSD-95 and β-actin. (**B**) Histograms showing relative expressions of PSD-95. White space was used to make explicit for the grouping of blots cropped from different parts of the same gel or from different gels. Experiments were repeated three times and data are shown as mean ± s.d. n = 3 rats per group. *p < 0.05, **p < 0.01, ***p < 0.001.

In order to examine the effect of imperatorin on synaptic damage in vascular dementia, we used transmission electron microscopy to observe the ultrastructure of hippocampal synapses in each group and examined the level of PSD-95 by Werstern blot. Our results show that compared with the Sham group, the hippocampal synapses in the VD rats were swollen, synaptic vesicles were sparse, synaptic space was blurred (Fig. 6A–E), synaptic active zone (SAZ) length was shortened (Fig. 6F), PSD thickness was thinned (Fig. 6G), and PSD-95 expression was reduced (Fig. 7). Compared with the VD group, IMP 2.5 mg/kg group showed synaptic structure was partially complete, synaptic vesicles, SAZ length, PSD thickness, and PSD-95 expression were increased. IMP 5 mg/kg and IMP 10 mg/kg group showed that the synaptic structure of hippocampus was relatively complete, with more synaptic vesicles, then SAZ length and PSD thickness were increased significantly, and PSD-95 expression was significantly up-regulated.

## Discussion

According to previous studies, apoptosis and synaptic damage caused by CCH are closely related to the onset of VD, especially damage to the hippocampus is more likely to cause cognitive decline, and then advance to VD^28^. At present, drugs for the clinical treatment of VD mainly target correlated risk factors, while drugs with excellent efficacy in cognitive function are still relatively lacking. Therefore, through establishing a reasonable and effective VD animal model to simulate the clinical pathogenesis of VD, actively exploring and discovering specific and effective drugs are of great value for further research on the occurrence and progress of VD. Our study suggested that IMP-treatment significantly reduced cognitive dysfunction caused by 2VO, attenuated the morphological damage of hippocampus CA1 pyramidal neuron. Its underlying molecular biological mechanism was mediated by suppressing neurons from apoptosis and reducing 2VO-incaused synaptic plasticity damage. These novel discoveries provide the pharmacological basis and mechanism of IMP for VD. Previous researches revealed that rats subjected to 2VO indicated a significant damage in learning and memory ability of hippocampus-dependent^29^. Similar to this results, the contemporary observations of MWM revealed that significant cognitive impairments appeared in 2VO rats. However, it obviously ameliorated the learning and memory impairment through the long-term treatment with IMP. In addition, HE staining showed that there were morphological injuries in hippocampus CA1 pyramidal neuron, and IMP-treatment reduced morphological defects in the hippocampus CA1 area, which was consistent with cognitive function results of the MWM. These findings obviously suggest that IMP has protective effects on the learning and memory impairment in rats with 2VO.

Neuronal apoptosis has been declared in 2VO rats, and Bcl-2 and Bax participate in the apoptosis signal transduction mechanism. When Bcl-2 is up-regulated, the Bcl-2/Bax heterodimer formed with Bax protein, which can act as a "molecular switch" on the mitochondrial membrane^30^, restricting the diffusion and diffusion of some apoptosis protein precursors outside the membrane, regulating the mitochondrial permeability transition pore opening degree, reducing ion channel activity, restricting some small molecules such as CytC from entering the cytoplasm, and ultimatley inhibiting apoptosis. The level of its expression will change the balance between pro-apoptotic genes and antiapoptotic genes, and is directly linked to whether cells undergo apoptosis^31^. Previous studies have confirmed that up-regulating the expression of Bcl-2 and reducing the expression of Bax can significantly limit the release of CytC, inhibit the activation of caspase-3 and the occurrence of apoptosis, and reduce cerebral ischemic injury. Furthermore, the degree of cognitive impairment is positively correlated with apoptosis^32^. Thus, strategies of the levels of Bax, Caspase-3 and/or promote the expression of Bcl-2 can reduce neuronal apoptosis, and can set-back 2VO induced cognitive impairment^33^. Similar to prior researches, the contents of Bax, Caspase-3 were significantly increased and the level of Bcl-2 was reduced after 2VO. However, IMP-treatment obviously reversed the level of Bax, Bcl-2, Caspase-3 and improved cognitive impairment in 2VO rats. Therefore, one of the molecular mechanisms by which IMP attenuates 2VO-induced cognitive impairments is linked to regulate the levels of apoptosis-related factors and inhibit neuronal apoptosis ultimately.

In addition, cognitive impairment after 2VO is strongly related to the damage of synapses and synaptic plasticity, especially synaptic morphology, interface structure and functional state. Studies have shown that PSD in the synaptic morphology has strong plasticity and extremely sensitive characteristics, and is susceptible to the afferent status of presynaptic nerve impulses and changes in the internal and external environment of the body, other synaptic interface structural parameters can vary with the function activity status changes of synaptic^34^. In the process of CCH, neurobiology has a series of reactions, such as cellular glucose utilization and body energy metabolism disorders, abnormal protein synthesis and delivery, etc. These pathological changes affect the ultrastructure of synapses and nerves, resulting in the degeneration and loss of synapses, and significant changes in synaptic structural parameters, such as the reduction of membrane surface before and after synapses, the reduction of SAZ length and PSD thickness, the fusion of synaptic clefts, etc. In severe cases, it may result in abnormal reconstruction of synaptic connections, that is, synaptic reconstruction^35^. Furthermore, PSD-95 is a key substance for post-synaptic signal transduction and integration. In different stages of CCH, its synaptic transmission, signal transduction and other related functions were impacted. In the early stage of cerebral ischemia, the expression of PSD-95 increased, which can enhance the transduction of glutamate excitatory signals overexpressed by NMDAR, and neuronal apoptosis; then with the prolongation of cerebral ischemia time, the level of PSD-95 decreased, which reduces the conduction of NMDAR in the learning and memory synaptic signaling pathway^17^. The changes in hippocampus synaptic structure parameters and the level of PSD-95 can reflect the synapse plasticity changes^36^. Consistent with previous findings, we found that the hippocampus anterior and posterior synaptic membranes of the 2VO rats were swollen, the number decreased, the synaptic gap was blurred, the synaptic vesicles were sparse, and vacuolated. Some typical synaptic structures disappeared, the length of SAZ was shortened, and the PSD was thinned, and the expression of PSD-95 was dramatically decreased. However, IMP treatment significantly improved synaptic ultrastructure morphology, and increased the SAZ length, PSD thickness and the level of PSD-95. These findings suggested that reduction of the synaptic plasticity damage caused by chronic cerebral ischemia and hypoxia is an important molecular mechanism of IMP against VD. Given its critical neuroprotective effect, further investigation into the use of IMP in clinical VD therapeutics is quite promising.

## Conclusion

We demonstrated that IMP attenuates 2VO-induced cognitive impairment and hippocampus neuron damage, and its underlying mechanism is likely due to regulate of pro- and antiapoptotic biomarkers, and improve hippocampus synapse ultra structural changes, increase SAZ length and PSD thickness, up-regulate the level of PSD-95. These findings provide a novel perspective on VD disease progression as well as demonstrate that IMP is a promising neuroprotective agent toward other neurodegenerative diseases.

## Supplementary Information

Supplementary Information.
