# Supplementary material for: Effects of imperatorin on apoptosis and synaptic plasticity in vascular dementia rats
Source: Sci Rep. 2021 Apr 21;11:8590. doi: 10.1038/s41598-021-88206-7 (PMC8060272; doi:10.1038/s41598-021-88206-7)
Supplement: Supplementary file 1 — Supplementary Information. [file 41598_2021_88206_MOESM1_ESM.docx]

**
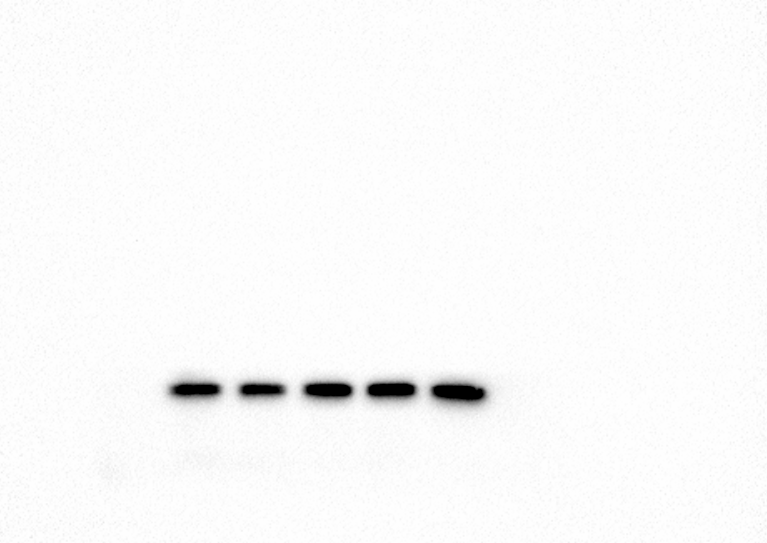

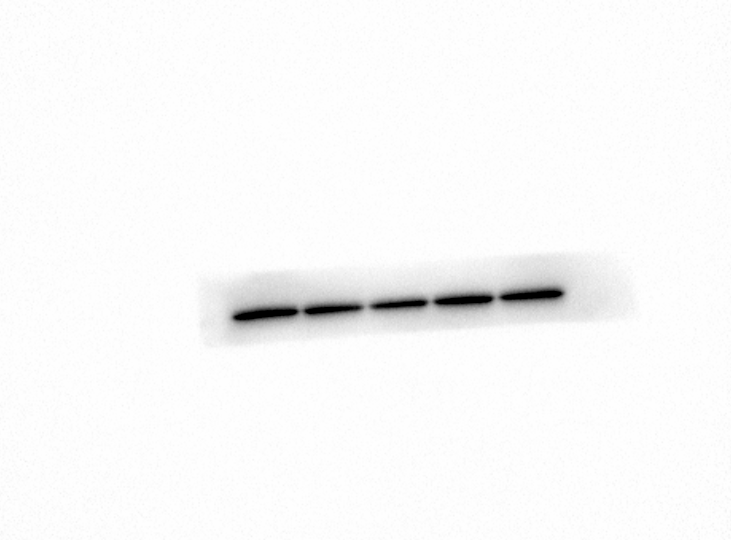

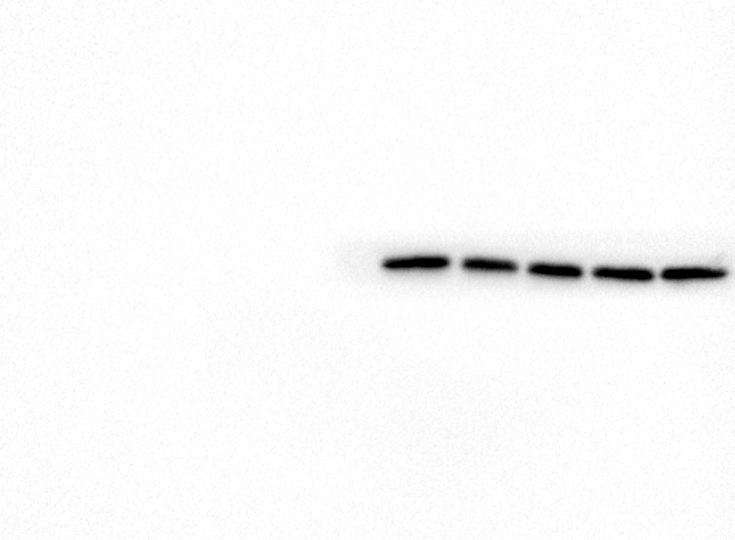
**

**
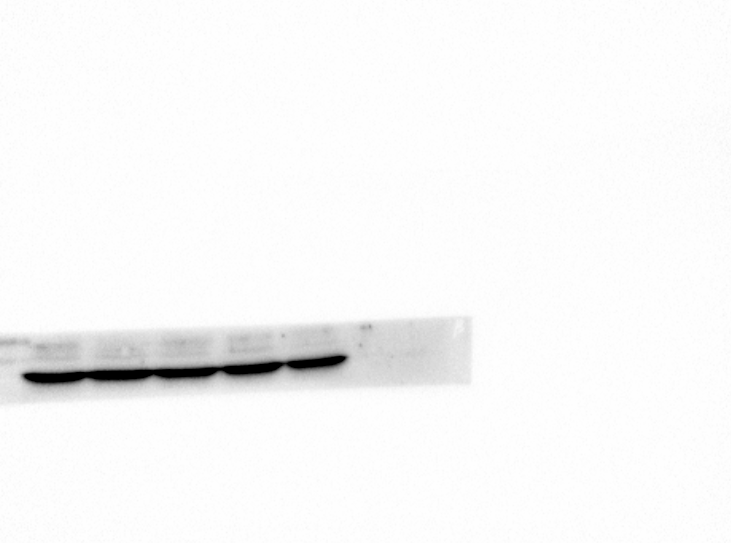

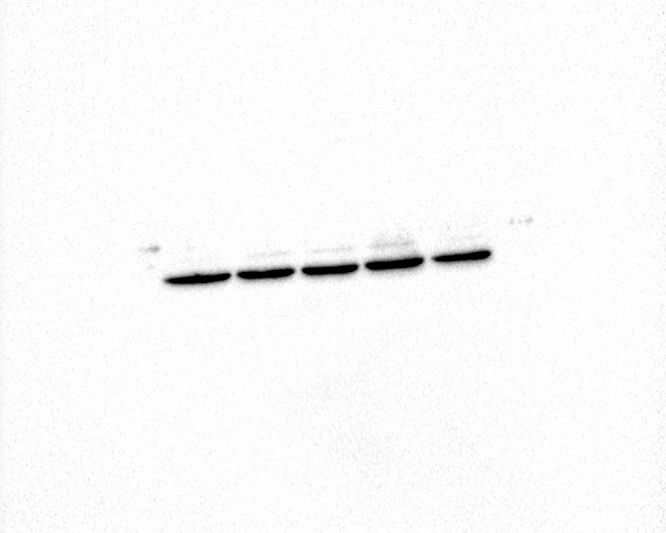

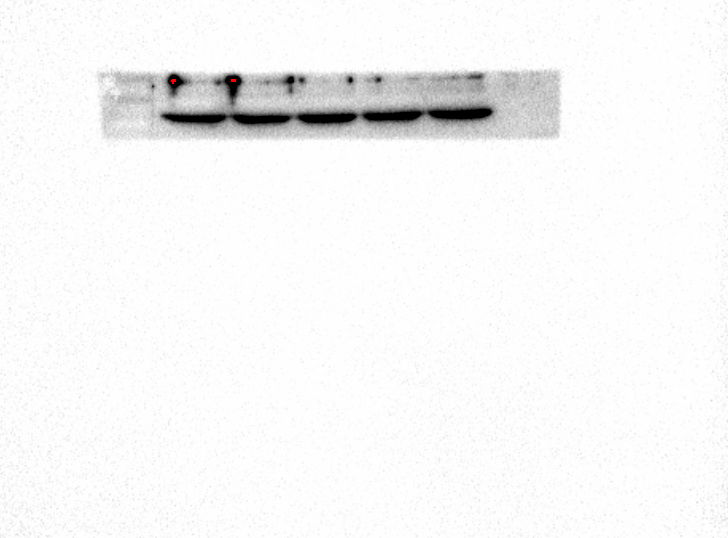
**

**the original image-Bcl-2/β-actin**

**
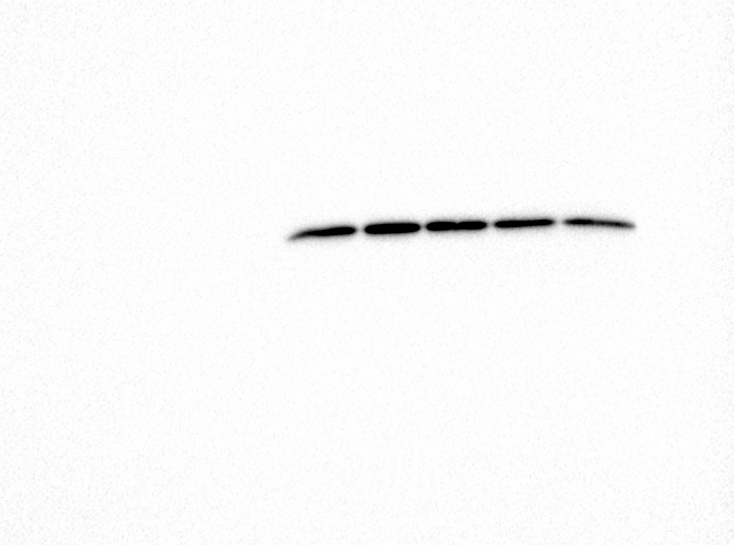

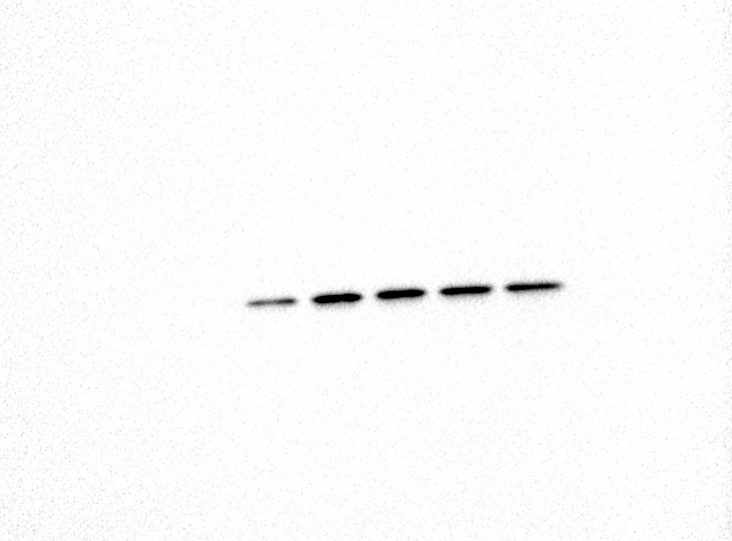

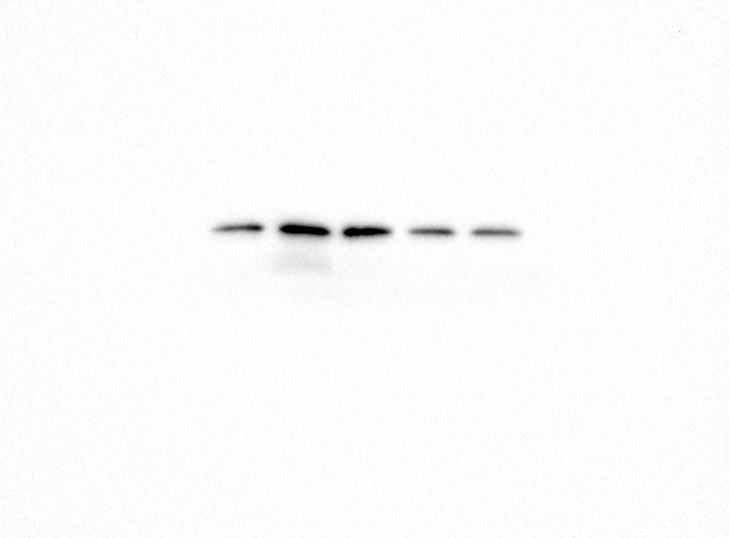

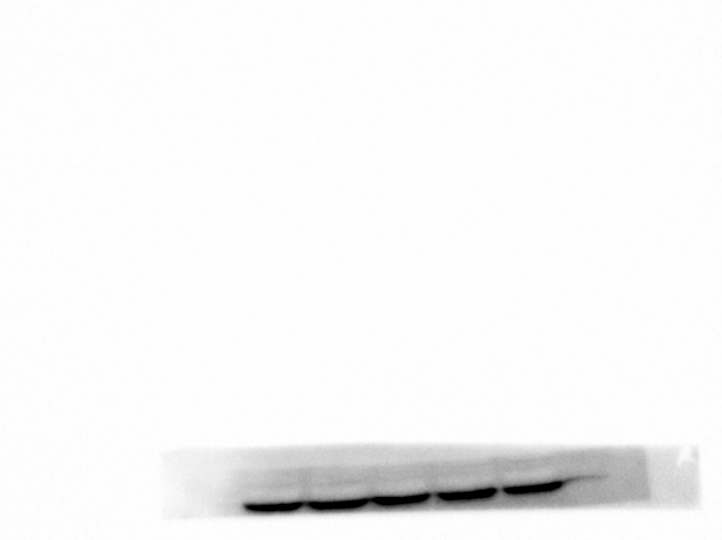

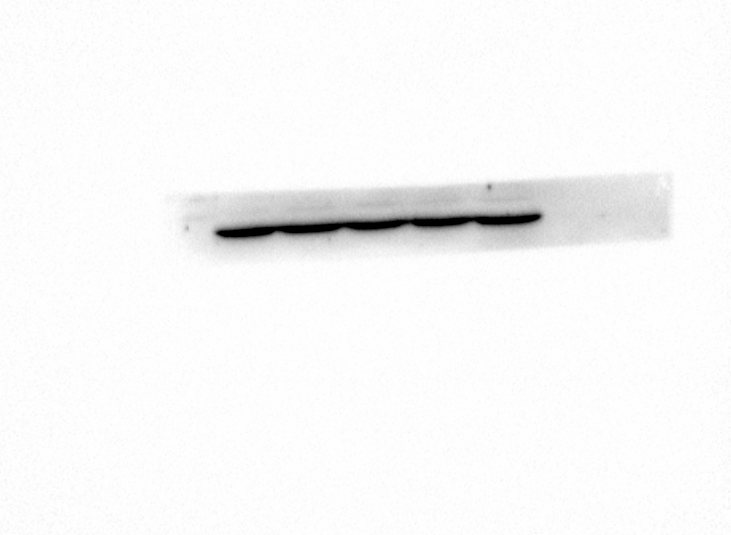

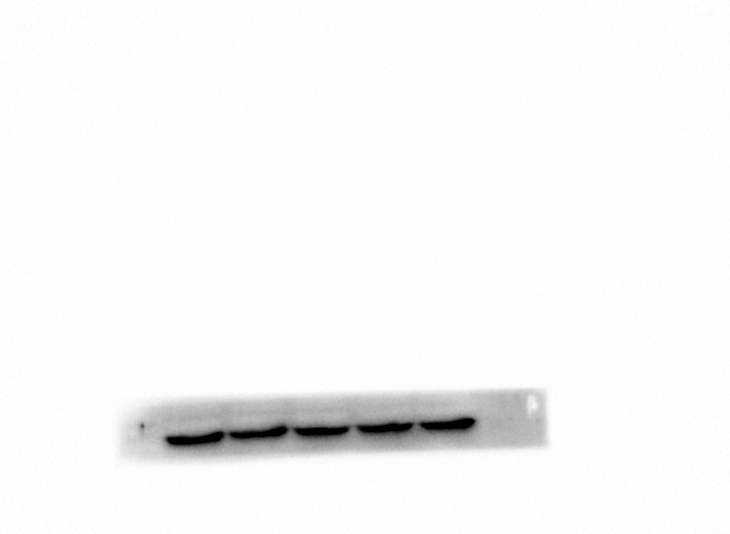
**

**the original image-Bax/β-actin**

**
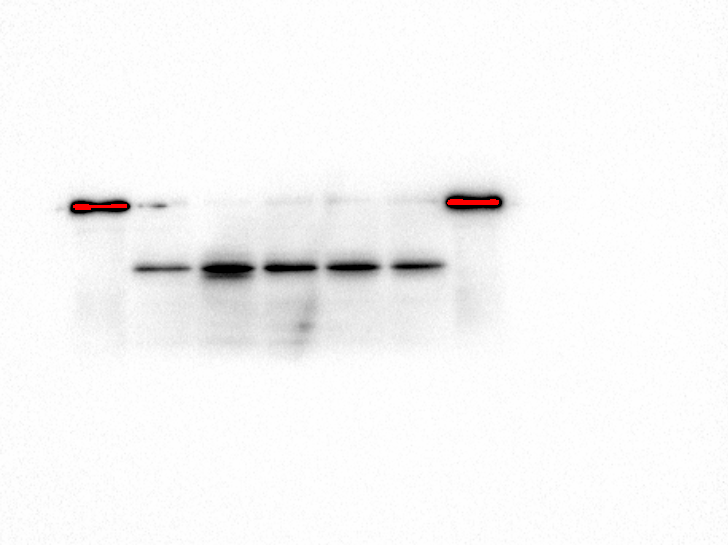

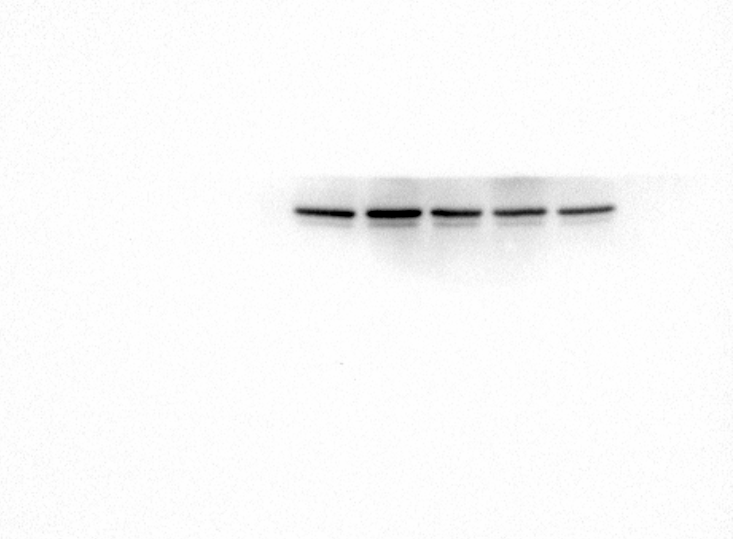

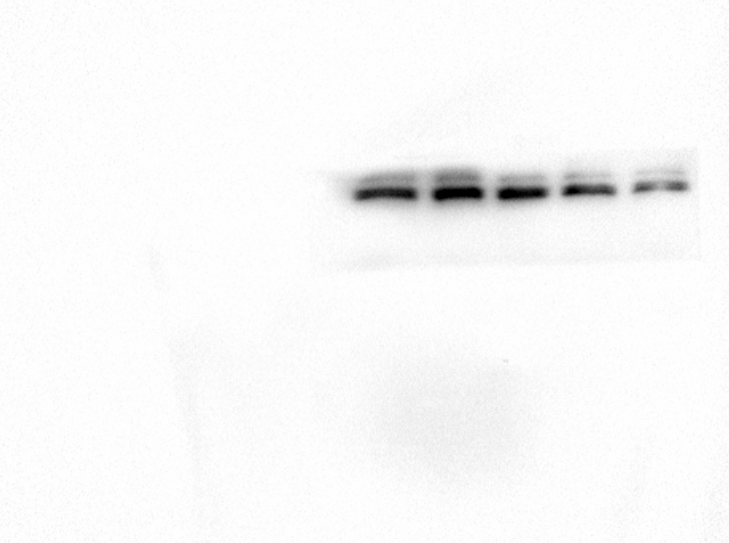
**

**
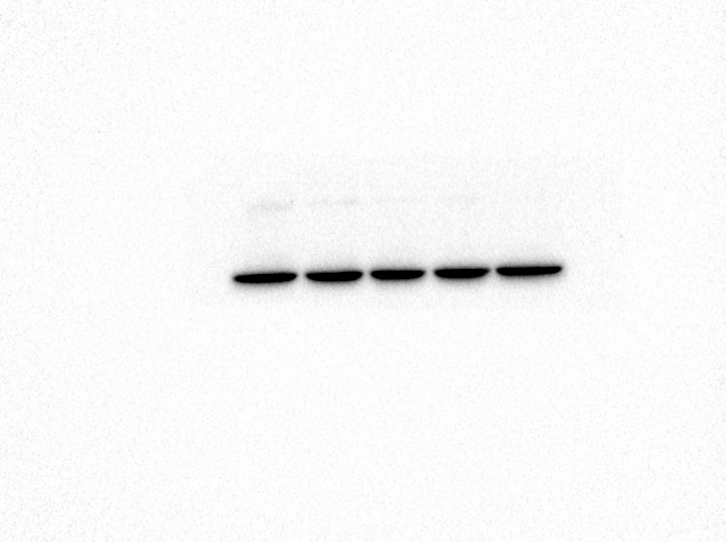

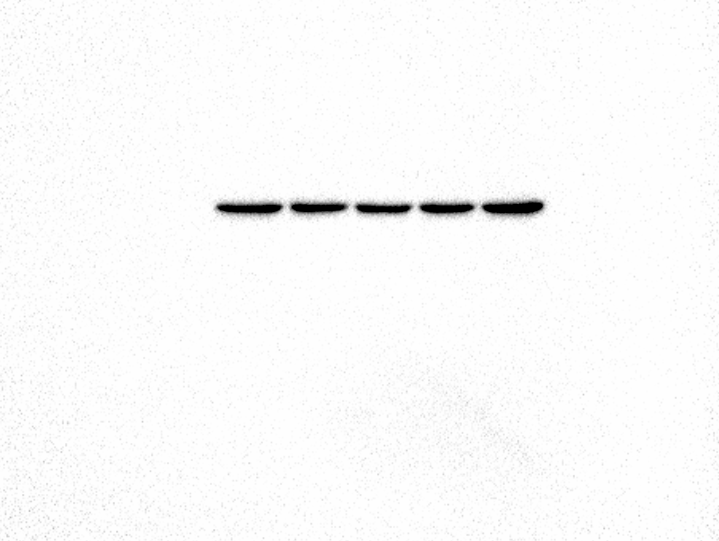

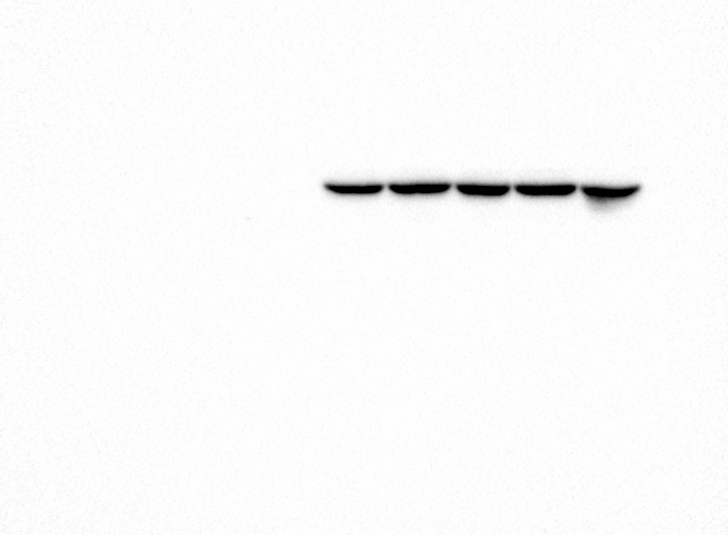
**

**the original image-Caspase-3/β-actin**

**
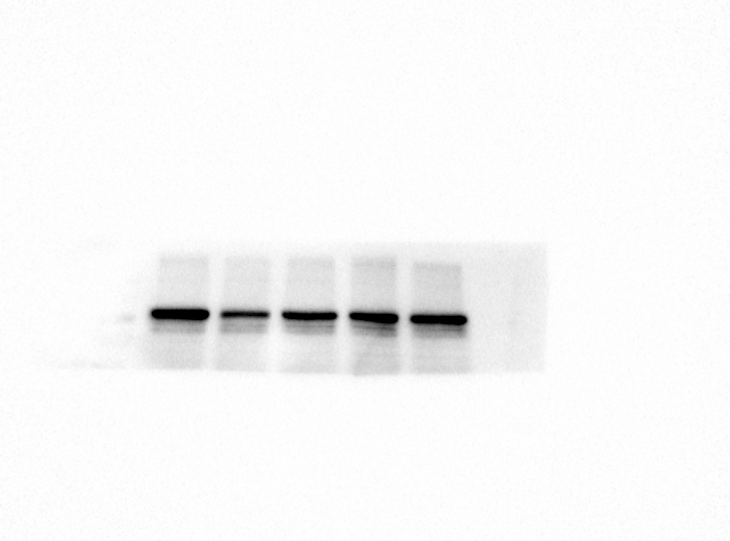

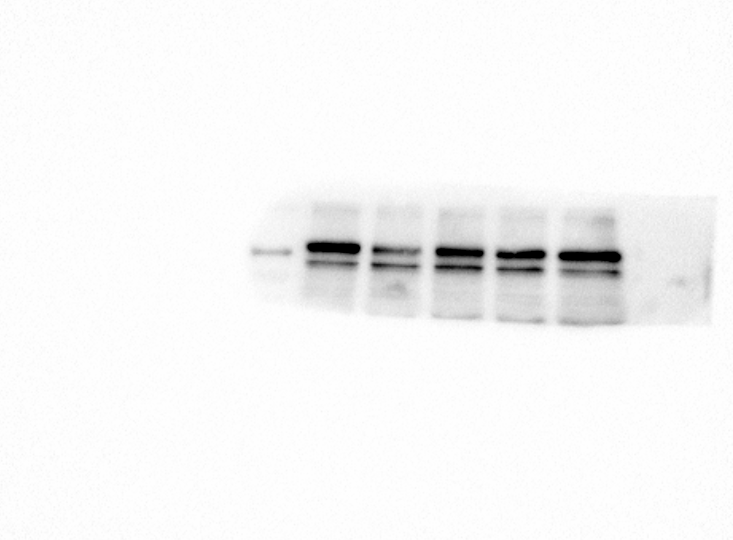

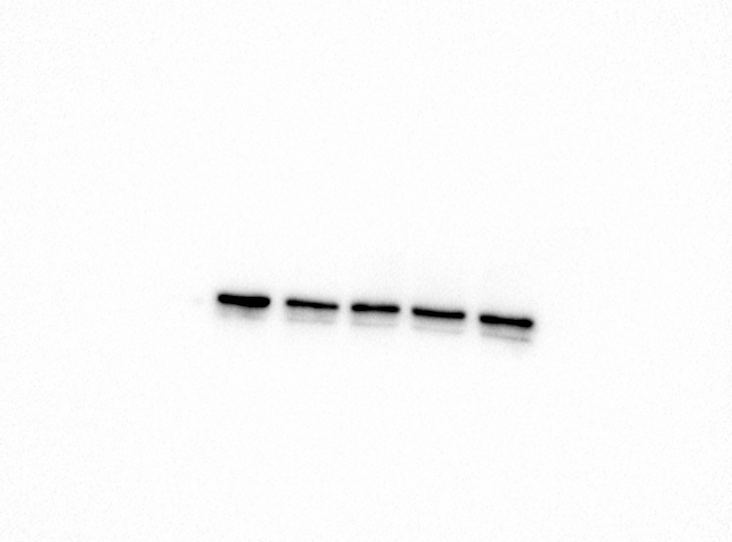

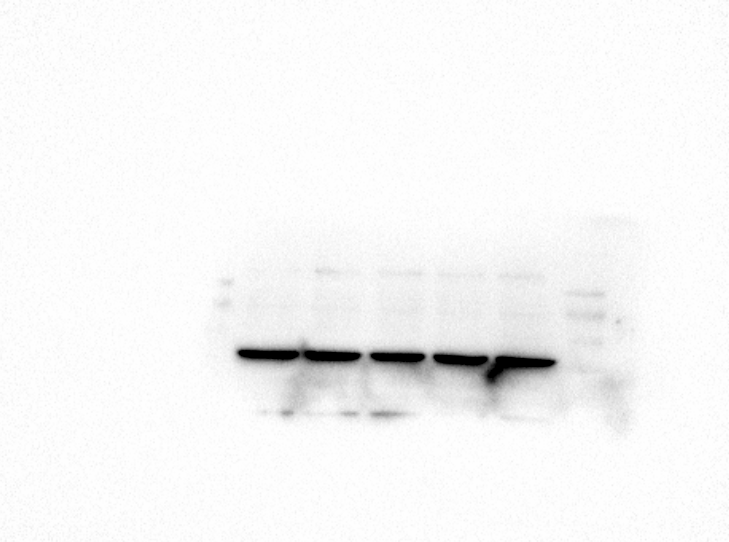

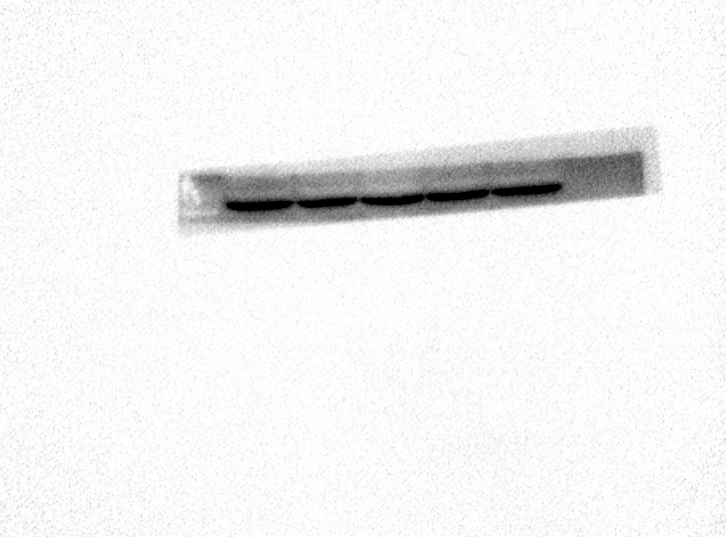

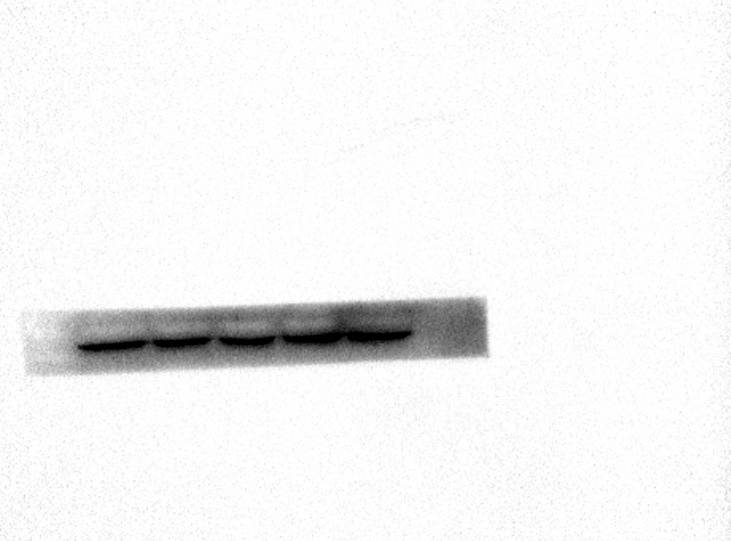
**

**the original image-PSD95/β-actin**
